# Long-Term Aging Study on the Solid State Interfacial Reactions of In on Cu Substrate

**DOI:** 10.3390/ma16186263

**Published:** 2023-09-18

**Authors:** Han-Tang Hung, Fu-Ling Chang, Chin-Hao Tsai, Chia-Yi Liao, C. R. Kao

**Affiliations:** Department of Materials Science & Engineering, National Taiwan University, Taipei 10617, Taiwan

**Keywords:** low melting-point solder, interfacial reaction, In, CuIn_2_, intermetallic compounds

## Abstract

Indium is considered a candidate low-temperature solder because of its low melting temperature and excellent mechanical properties. However, the solid-state microstructure evolution of In with different substrates has rarely been studied due to the softness of In. To overcome this difficulty, cryogenic broad Ar^+^ beam ion polishing was used to produce an artifact-free Cu/In interface for observation. In this study, we accomplished phase identification and microstructure investigation at the Cu/In interface after long-term thermal aging. CuIn_2_ was observed to grow at the Cu/In interface and proved to be a stable phase in the Cu–In binary system. The peritectoid temperature of the Cu_11_In_9_ + In → CuIn_2_ reaction was confirmed to be between 100 and 120 °C. In addition, the growth rate of CuIn_2_ was discovered to be dominated by the curvature of the reactant Cu_11_In_9_/In phase and the temperature difference with the peritectoid temperature. Finally, a comprehensive microstructural evolution mechanism of the Cu/In solid-state interfacial reaction was proposed.

## 1. Introduction

With the rapid growth in wearable technology and the internet of things (IoT), low-temperature bonding has been investigated intensively over the years because it can prevent the risk of bond failure induced by heat localization at sensors, actuators, optoelectronic devices, and advanced microelectromechanical systems (MEMSs) embedded in IoT systems [1,2]. Among numerous low-temperature bonding strategies, solid-liquid interdiffusion (SLID) bonding [3], also called transient liquid-phase (TLP) bonding [4], is the most reliable bonding technique and is broadly utilized in electronic packaging at present. The SLID bonding technique is based on a binary interlayer structure, including a high melting-point substrate metal and a low melting-point solder alloy. When the temperature rises above the melting point of the solder, the molten solder reacts with the solid substrate and forms intermetallic compounds (IMCs), which have relatively high melting points. Therefore, without residual solder in the joint after bonding, the SLID process not only has the advantage of low-temperature bonding but also enables further high-temperature applications. However, it should be noted that there is a limit to how low the reflow temperature can go, which is the melting point of the solder alloy employed. To that end, since the melting points of the widely used high-Sn Pb-free alloys at present are high for heat-sensitive materials, the development of new solder alloys with low melting points to reduce the reflow temperature of the SLID process is essential.

Over recent years, pure indium has been successfully employed as a low-temperature soldering material by Panchenko et al., and fine-pitch Cu–In bonding based on the SLID process was carried out at a peak temperature of 170 °C for 2 min [5,6,7,8]. In their research, the Cu/In joints revealed remarkable shear strengths, ranging from 45 to 120 MPa [7], which were comparable with the strengths of Cu/Sn interconnects [9]. Accordingly, In was a potential material candidate for low-temperature SLID bonding and thermal interface materials [10,11,12,13,14]. Fundamental information on the interfacial reactions between In and Cu is crucial for evaluating the feasibility of In solder. Our recent study revealed the interfacial reactions between liquid In and solid Cu at 180 °C [15]. A two-phase layer consisting of Cu_11_In_9_ and In and faceted Cu_11_In_9_ particles over the two-phase layer formed at the Cu/In interface [15].

**Figure 1:**
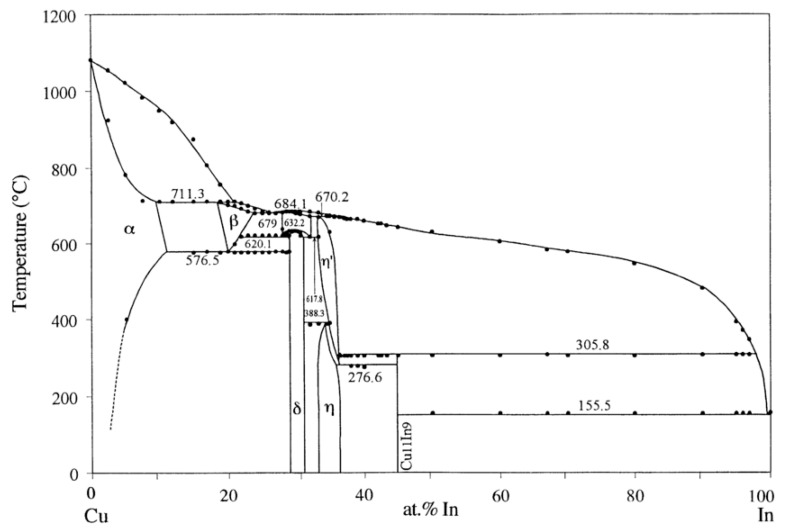
The latest published Cu–In phase diagram [24].

With regard to the solid-state interfacial reactions between In and Cu, a layer of CuIn_2_ was discovered at the interface of sequentially evaporated Cu/In thin films [16,17,18,19,20,21,22] and as-electroplated Cu/In films [6,7,23] at room temperature. Although the CuIn_2_ phase was not included in the latest equilibrium Cu–In phase diagram as shown in Figure 1 [24], its formation in bulk Cu–In [25,26] and Cu–In–S [25,27] alloys after quenching indicated that it is a stable phase at room temperature and is likely formed through the peritectoid reaction between Cu_11_In_9_ and In [26]. Nonetheless, the temperature stability of CuIn_2_ has not yet been determined, since the decomposition temperature of CuIn_2_ reported in different studies varied from 107 °C [17] to 130 °C [25,28] and 148 °C [19]. Recently, Lin et al. discussed the interfacial reactions of electroplated Cu/In films from 100 to 180 °C [23]. Their results suggest that the peritectoid temperature of CuIn_2_ lies in the region between 100 and 120 °C, and Cu_11_In_9_ is the dominant phase when the reaction temperature is higher than 120 °C. In summary, previous studies have tended to investigate solid-state Cu/In interfacial reactions in the thin-film case to avoid the polishing difficulties introduced by the soft In phase [6,16,17,18,19,20,21,22,23,28,29], and there is a literature gap regarding the interfacial reactions between solid In and Cu in the bulk case. In addition, previous studies ignored the fact that the interfacial reactions between In and Cu were still taking place during room-temperature storage, leading to the erroneous determination of the temperature stability of CuIn_2_. Thus, this paper aims to fill the gap in the literature by providing a comprehensive study.

In the present study, we investigated the interfacial reactions between solid In and Cu in the bulk case. Because long-term annealing at low temperature is required to reach equilibrium, the annealing times in this study ranged from 12 h to more than 5000 h. Moreover, the duration of sample storage at room temperature was recorded to prevent the inaccurate determination of the microstructure evolution. A cryogenic broad argon ion milling technique was employed to provide artifact-free cross-sectional microstructures. The microstructure evolution of the interfacial reaction between solid In and Cu and the peritectoid temperature of the CuIn_2_ phase were then studied and discussed.

## 2. Experimental Procedures

**Figure 2:**
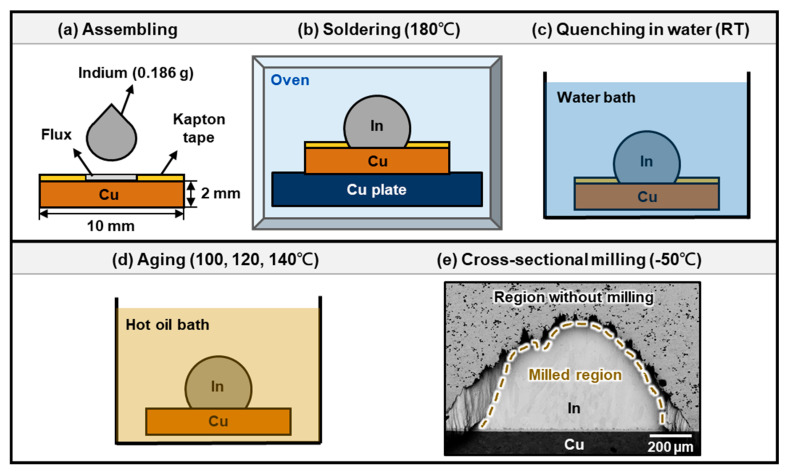
Schematic drawings showing the experimental procedures in this research.

The sample preparation and experimental procedures are illustrated in Figure 2. The copper substrates used in this study were prepared by mechanically grinding and polishing 99.99% oxygen-free Cu plates (Goodfellow, Huntingdon, UK), which measured 10 mm × 10 mm × 2 mm. The last step of the polishing was made with a 1 μm Al_2_O_3_ suspension to achieve a mirror-like copper surface. These Cu substrates were etched with 5% H_2_SO_4_ solution for 1 min and then cleaned with deionized water for 3 min to remove the copper oxide. Kapton tape with an opening diameter of 4 mm in the middle was then applied to each of the Cu substrates as a solder mask, and a halogen-free no-rinse solder flux was pasted over the holes in the Kapton tape on the Cu substrates, as shown in Figure 2a. After this, an indium shot of 99.9995% purity (Indium Corporation, Clinton, NY, USA) with a weight of 0.186 g was placed on each Cu substrate as the solder. The assembled samples were placed in a convection oven at 180 ± 1 °C for 30 s for soldering, as illustrated in Figure 2b. The process of soldering was carried out to ensure reliable contact of In with Cu. The samples were then retrieved from the oven and quenched in water, as illustrated in Figure 2c. Subsequently, the water-quenched samples were aged in hot oil baths at 100, 120, and 140 °C with a temperature stability of ±1 °C for periods up to 5000 h, as shown in Figure 2d. To elucidate the effect of water quenching and room temperature storage on the formation of CuIn_2_, some of the samples were directly aged in oil baths without a water-quenching process or additionally stored at room temperature for a long period after aging in the oil bath.

For each specific aging time, the samples were mounted in epoxy resin, sectioned by using a low-speed diamond saw, and metallographically ground and polished down to 1 μm diamond powder suspensions. To address the problem of the embedment of the SiC and diamond abrasives in the soft indium phase during conventional mechanical grinding and polishing, additional argon ion milling polishing (IM4000Plus, Hitachi, Tokyo, Japan) was used for final polishing to obtain artifact-free cross-sectional samples. In addition, because of the low melting point of indium, a cryogenic liquid nitrogen cooling unit was utilized to provide a constant low temperature during ion milling. Figure 2e shows the cross-section of the Cu/In interface after ion milling at −50 °C for 2 h. It can be seen that the milled region presented no artifact from mechanical polishing.

Afterward, the morphology of the interfacial layers and the microstructure evolution of the intermetallic compounds at the interface were examined by a scanning electron microscope (SEM, Hitachi, SU5000) equipped with a backscattered electron detector. Eventually, energy-dispersive X-ray spectroscopy (EDXS) was utilized for compositional analysis.

## 3. Results and Discussion

### 3.1. Evolution of the Microstructure during Thermal Aging

**Figure 3:**
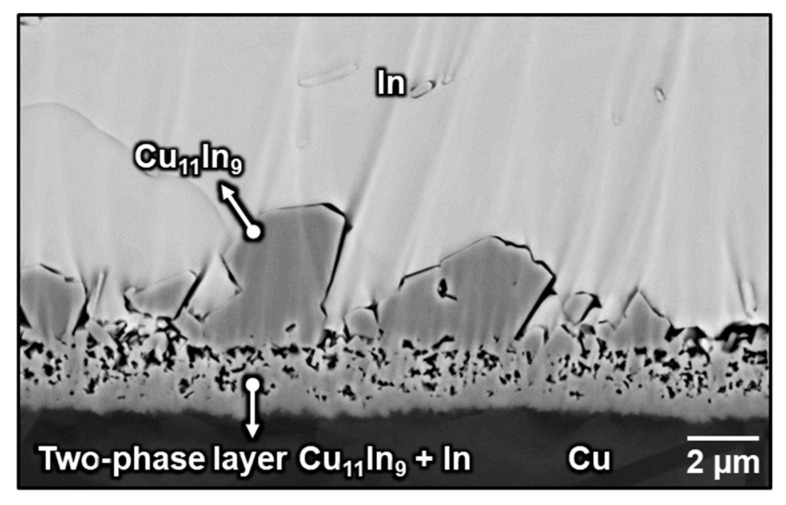
Microstructure of the Cu/In interface after soldering at 180 °C for 30 s. This figure is adopted from our previous study [10].

Figure 3 shows the cross-sectional microstructure of the as-bonded Cu/In interface after soldering at 180 °C for 30 s followed by water quenching. Consistent with our previous study [15], a two-phase layer and some heterogeneously precipitated faceted Cu_11_In_9_ particles formed at the Cu/In interface. The two-phase layer comprised Cu_11_In_9_ and In, where In corresponded to the dark areas produced when argon gas removed the soft In surrounded by tough Cu_11_In_9_ during ion polishing.

**Figure 4:**
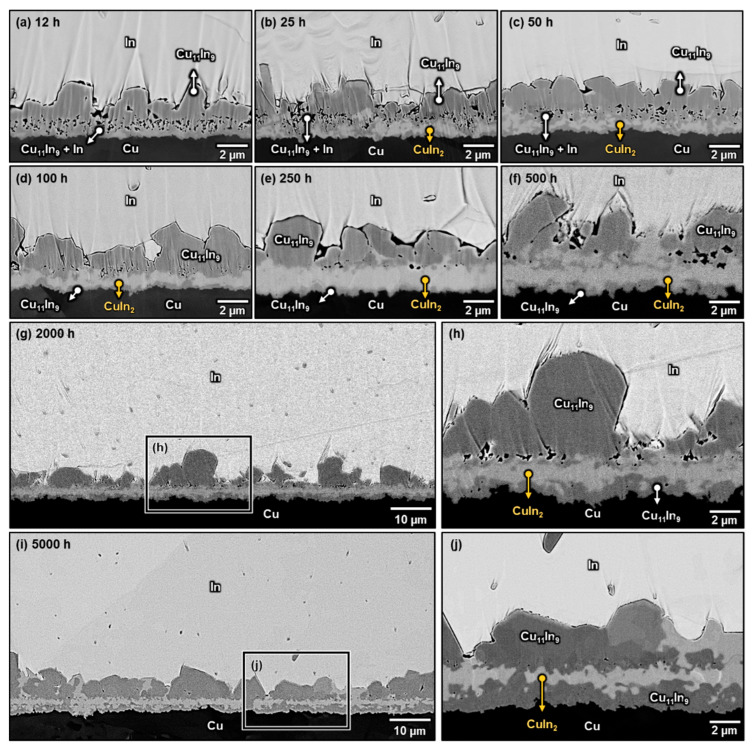
Micrographs showing the Cu/In interfaces after soldering at 180 °C for 30 s, followed by aging at 100 °C for (**a**) 12 h, (**b**) 25 h, (**c**) 50 h, (**d**) 100 h, (**e**) 250 h, (**f**) 500 h, (**g**) 2000 h, and (**i**) 5000 h. (**h**,**j**) are the enlarged micrographs of the rectangle in (**g**,**i**).

Figure 4a–j shows the microstructures of the Cu/In interfaces after aging at 100 °C for 12–5000 h. The CuIn_2_ phase was formed in the two-phase layer and coarsened gradually with time. After 250 h, CuIn_2_ coarsened into a continuous layer, and no Cu_11_In_9_ and In remained in the original two-phase layer region, implying that the formation of CuIn_2_ was probably based on the peritectoid reaction from Cu_11_In_9_ and In in the two-phase layer. Additionally, the result that CuIn_2_ had not disappeared after aging for 5000 h indicated that it is an equilibrium phase at 100 °C. However, it is interesting that although the attached Cu_11_In_9_ particles were also surrounded by a large amount of In, they only increased in size, and no CuIn_2_ was formed at the interface between them and the In matrix even after long-term aging at 100 °C. The reason for this has been reported by Klinger et al. [30]. They discussed the peritectoid reaction *α* + *β* → *ω* specialized to the case of *ω* growth along the *α*/*β* interface at comparatively low temperatures in which volume diffusion is negligible and interphase boundary diffusion is rate-controlling. With the assumption that the diffusion along the interfaces is governed by the gradients of interfacial curvature, they described the chemical driving force (Δ*G*_0_) and the thickness (*H*) and lengthening velocity (*V*) of *ω* through the following equations [30]:

(1) $$∆G_{0}=\Omega \cdot [\frac{c_{\beta }-c_{\omega }}{c_{\beta }-c_{\alpha }}\cdot \gamma_{\alpha \omega }\cdot K_{\alpha \omega }+\frac{c_{\alpha }-c_{\omega }}{c_{\alpha }-c_{\beta }}\cdot \gamma_{\beta \omega }\cdot K_{\beta \omega }]$$

(2) $$H=\frac{2\Omega \cdot ∆\gamma }{∆G_{0}}$$

(3) $$V=[\frac{∆G_{0}\;\left(c_{\beta }-c_{\alpha }\right)}{\left(c_{\beta }-c_{\omega }\;\right)\;\left(c_{\omega }-c_{\alpha }\right)\;\Omega \;{\left(2∆\gamma \right)}^{1/2}}{]}^{3}\cdot {\left[\left(\frac{\gamma_{\alpha \omega }{}^{1/2}}{L_{\alpha \omega }\;\Omega \;\left(c_{\omega }-c_{\alpha }\right)}\right)\right]}^{2/3}+{\left(\frac{{\left(\gamma_{\beta \omega }\right)}^{1/2}}{L_{\beta \omega \;}\Omega \;\left(c_{\beta }-c_{\alpha }\right)}\right)}^{2/3}{]}^{3/2}$$

where Ω are the atomic volumes in the various phases, which are assumed to be identical, *γ* is the surface energy of the interface, Δ*γ* is the surface tension gradient (Δ*γ* = *γ_αω_ + γ_βω_* − *γ_αβ_*), *K* is the curvature of the interface, *c* is the mole fraction of one of the two elements, and *L* is the mobility of atoms along the interface. Therefore, when the other parameters are constant, the curvature of the *α*/*β* interface plays an important role in determining the chemical driving force of *ω* formation and further influences the layer thickness and the lengthening velocity of *ω*. Accordingly, it can be deduced that the absence of CuIn_2_ between the attached Cu_11_In_9_ particles and the In matrix was due to the relatively flat interface between them, which decreased the driving force for the reaction and slowed the lengthening velocity. Furthermore, it can be noted that when the original two-phase layer gradually transformed into a continuous CuIn_2_ layer, several Cu_11_In_9_ islands were formed between the continuous CuIn_2_ layer and Cu, as shown in Figure 4b–f. These islands gradually joined together and became a thick and continuous Cu_11_In_9_ layer after 2000 h of aging, as illustrated in Figure 4h,j. Since the original Cu/In interface was between the faceted Cu_11_In_9_ particles and the two-phase layer [15], the growth front of the continuous Cu_11_In_9_ layer was observed to be at the Cu side, indicating that In was the dominant diffusion species in the Cu/In system at 100 °C. Accordingly, In atoms diffused across the continuous CuIn_2_ and Cu_11_In_9_ layers to react with Cu and formed new Cu_11_In_9_.

**Figure 5:**
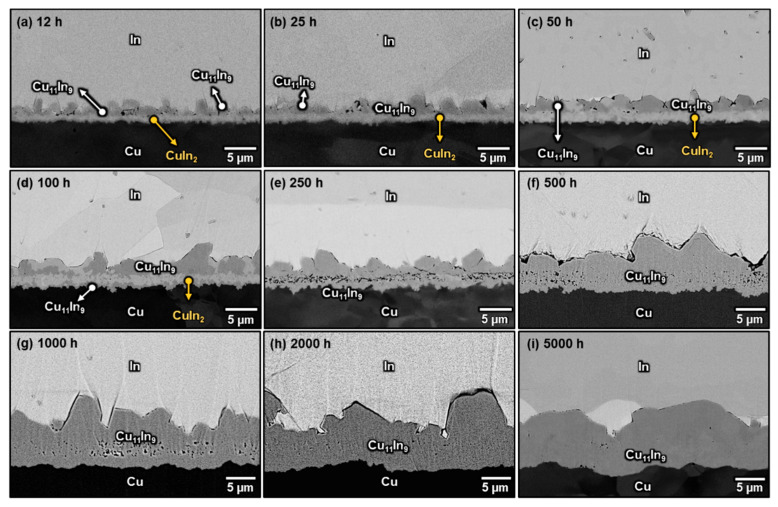
Micrographs showing the Cu/In interfaces after soldering at 180 °C for 30 s, followed by aging at 120 °C for (**a**) 12 h, (**b**) 25 h, (**c**) 50 h, (**d**) 100 h, (**e**) 250 h, (**f**) 500 h, (**g**) 1000 h, (**h**) 2000 h, and (**i**) 5000 h.

**Figure 6:**
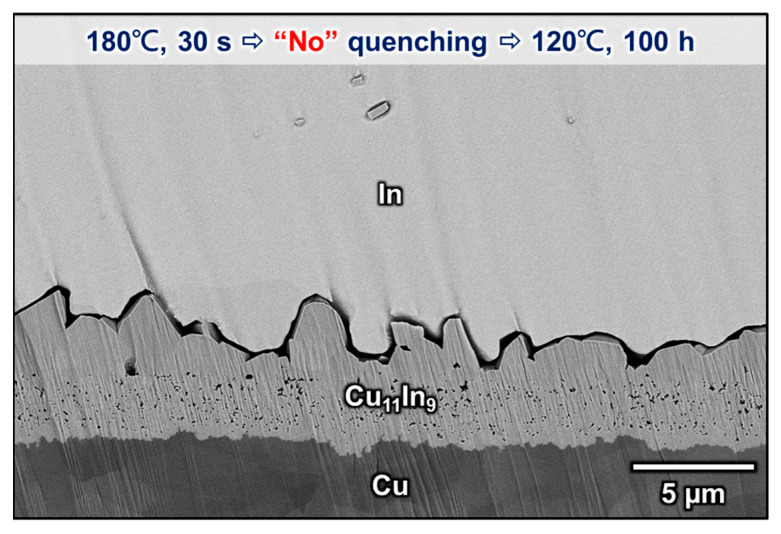
Microstructure of the Cu/In interface after soldering at 180 °C for 30 s and direct aging at 120 °C for 100 h.

Figure 5a–i shows the microstructures of the Cu/In interfaces after solid-state aging at 120 °C for 12–5000 h. When the aging time was less than 100 h, the CuIn_2_ phase was observed in the two-phase layer. However, after 250 h of aging, CuIn_2_ decomposed into Cu_11_In_9_ and In, and the microstructure became a continuous layer of Cu_11_In_9_ with embedded discontinuous In islands, indicating that CuIn_2_ is not an equilibrium phase at 120 °C. When the aging time was prolonged to more than 500 h, all the residual In in the continuous Cu_11_In_9_ layer had completely disappeared, and the interfacial structure had transformed into a layer-type morphology. The phenomenon of CuIn_2_ appearing and then disappearing at the interface when aging at 120 °C was probably caused by the water-quenching process between soldering and aging, because the long-term aging results revealed that CuIn_2_ is not a stable phase at 120 °C. To test this conjecture, the other sample was directly dipped in a hot oil bath at 120 °C for 100 h after soldering at 180 °C for 30 s without a water-quenching process. As shown in Figure 6, Cu_11_In_9_ was the only compound formed at the Cu/In interface during aging at 120 °C. Thus, the formation of CuIn_2_ observed in Figure 5a–d was likely because these specimens were quenched in water and stayed at room temperature for several minutes and because CuIn_2_ took more than 100 h to decompose at 120 °C.

**Figure 7:**
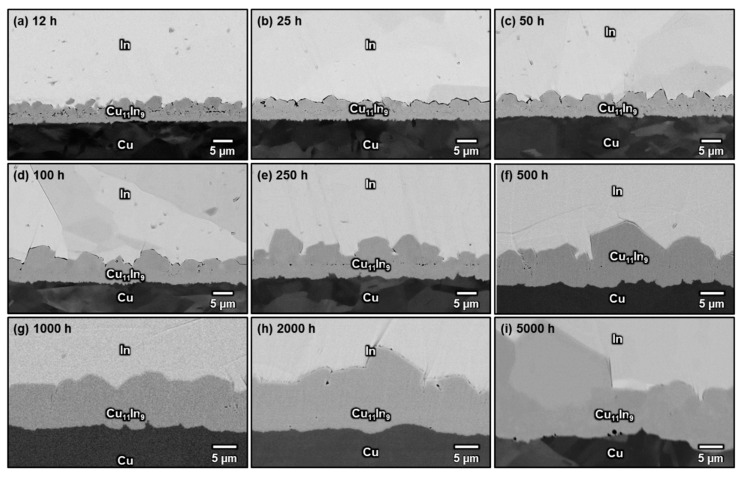
Micrographs showing the Cu/In interfaces after soldering at 180 °C for 30 s, followed by aging at 140 °C for (**a**) 12 h, (**b**) 25 h, (**c**) 50 h, (**d**) 100 h, (**e**) 250 h, (**f**) 500 h, (**g**) 1000 h, (**h**) 2000 h, and (**i**) 5000 h.

**Figure 8:**
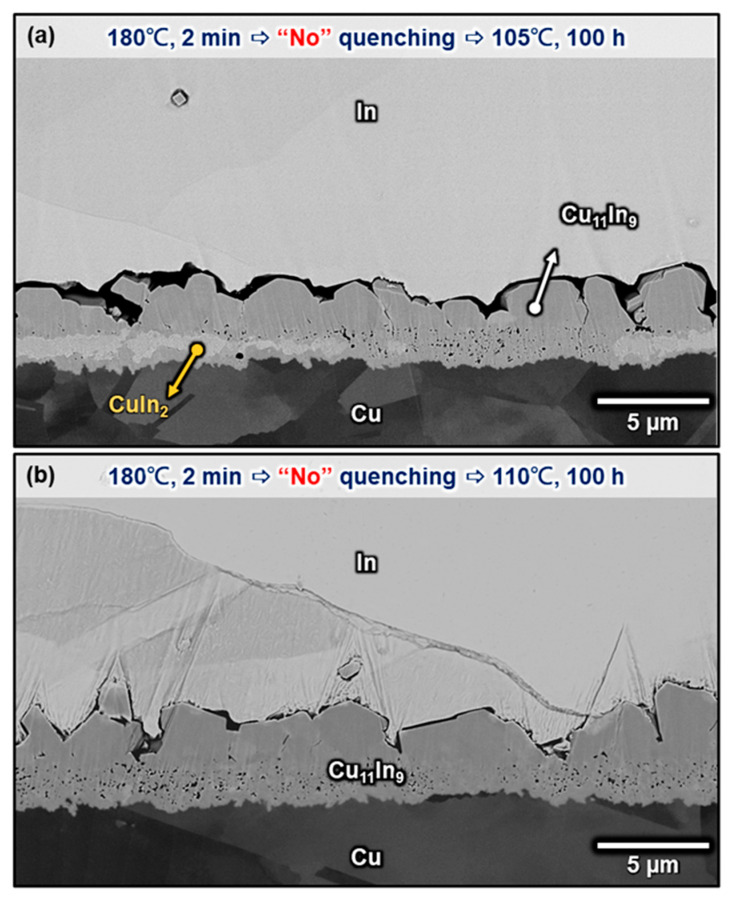
Micrographs showing the Cu/In interfaces after soldering at 180 °C for 2 min and direct aging at (**a**) 105 °C and (**b**) 110 °C for 100 h.

Figure 7a–i shows the microstructures of the Cu/In interfaces after solid-state aging at 140 °C for 12–5000 h. Although these samples were also quenched in water and stayed at room temperature for a couple of minutes, no CuIn_2_ was observed even after merely 12 h of aging, and the interfacial microstructure quickly became a thick and continuous layer of Cu_11_In_9_. This is explained in terms of the relatively faster diffusion kinetics and reaction kinetics at 140 °C. Based on these results, it is suggested that there exists a peritectoid reaction Cu_11_In_9_ + In → CuIn_2_ between 100 and 120 °C, which is consistent with our previous study [23]. Figure 8 demonstrate the samples that were directly immersed in an aging oil bath at the targeted temperature when they were retrieved from the 180 °C oven. CuIn_2_ only formed at an aging temperature of 105 °C but not at 110 °C, indicating that the peritectoid temperature of CuIn_2_ may be between 110 and 105 °C. However, a more accurate CuIn_2_ decomposition temperature remains to be analyzed more comprehensively.

### 3.2. Solid-State Aging at Room Temperature

**Figure 9:**
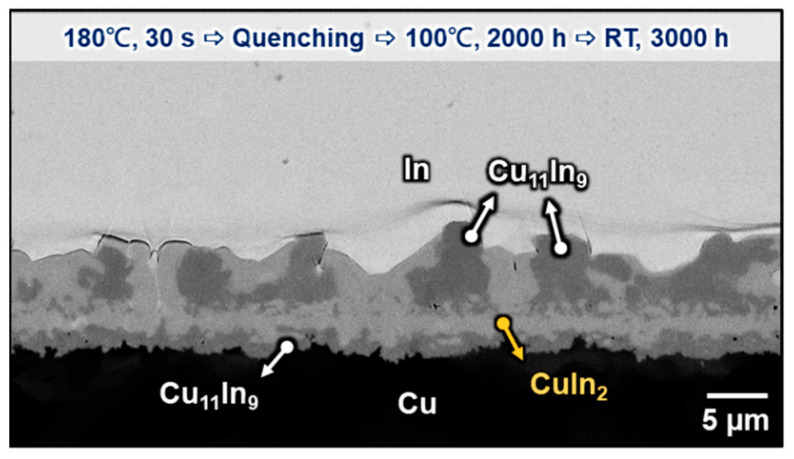
Micrograph showing the Cu/In interfaces after soldering at 180 °C for 30 s, followed by aging at 100 °C for 2000 h and restoring at room temperature for 3000 h.

**Figure 10:**
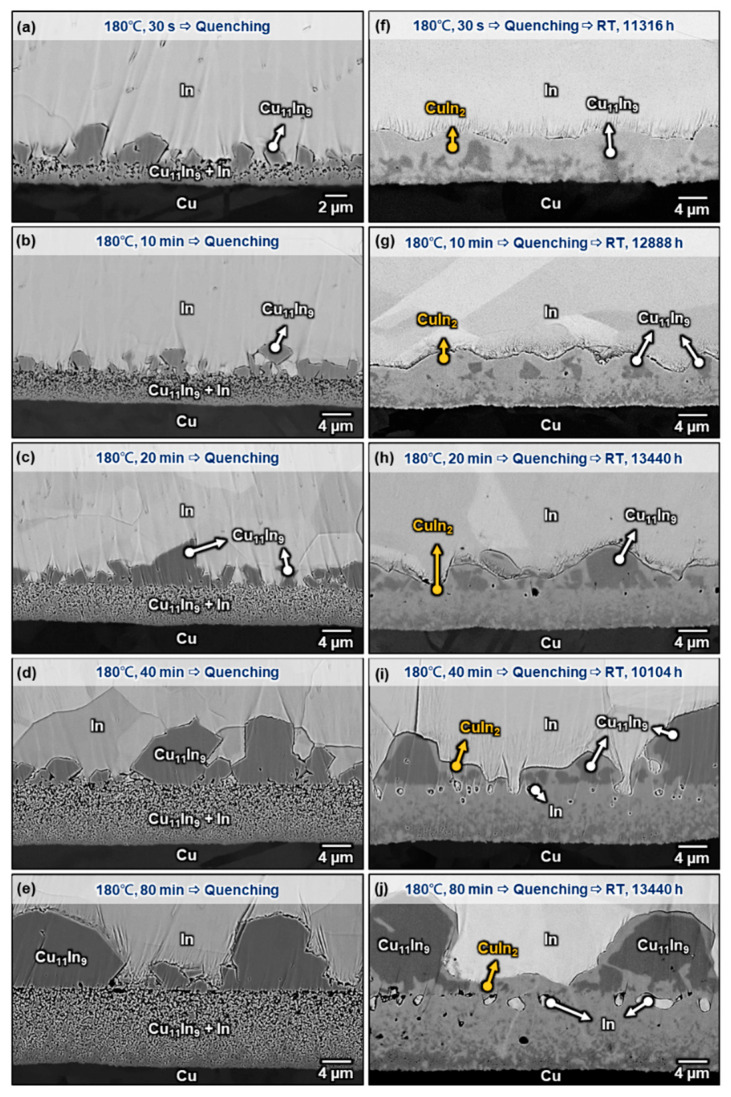
Micrographs showing the Cu/In interfaces after soldering at 180 °C for (**a**) 30 s, (**b**) 10 min, (**c**) 20 min, (**d**) 40 min, and (**e**) 80 min, and (**f**–**j**) successively stored at room temperature for more than 10,000 h. (Source (**a**–**e**) are adopted from our previous study [10]).

Because the interfacial reaction between In and Cu can be carried out at room temperature, the samples studied above were stored at room temperature for thousands of hours until further analysis. Figure 9 shows the microstructure of the Cu/In interface after aging at 100 °C for 2000 h plus 3000 h at room temperature. Compared with the specimens without room temperature storage, as shown in Figure 4g–j, the continuous CuIn_2_ layer, which was formed in the two-phase layer during 100 °C thermal aging, propagated along the grain boundary of the attached Cu_11_In_9_ particles at room temperature, resulting in these Cu_11_In_9_ particles being encapsulated by CuIn_2_ from the bottom up. Similar phenomena can also be found in the samples without thermal aging. Figure 10a–e are the cross-sectional micrographs of the Cu/In interfaces after soldering at 180 °C for 30 s, 10 min, 20 min, 40 min, and 80 min. (reproduced from our previous study [10]) When these structures were stored at room temperature straight after 180 °C soldering for more than 10,000 h, it can be seen from Figure 10f–j that the as-formed two-phase layer after quenching was transformed into a continuous CuIn_2_ layer with some embedded In particles on the side near In and a dendritic Cu_11_In_9_ layer on the side near Cu. This distribution of composition is speculated to be caused by the high In content on the side near the In matrix and the high Cu_11_In_9_ content on the Cu side in the as-formed two-phase layer. The Cu_11_In_9_ particles attached to the continuous layer were surrounded by CuIn_2,_ which was presumed to grow from the continuous layer below and along the interphase boundary between the attached Cu_11_In_9_ particles and the In matrix. The results above reconfirmed the aforementioned Equation (3) that the lengthening velocity (*V*) is found to depend on the cube of the driving force (Δ*G*_0_)^3^ when volume diffusion is negligible and interphase boundary diffusion is rate-controlling at room temperature [30]. In other words, the lower the temperature and the larger the driving force, the faster this peritectoid reaction is likely to be, especially for the interphase with a relatively flat interface.

**Figure 11:**
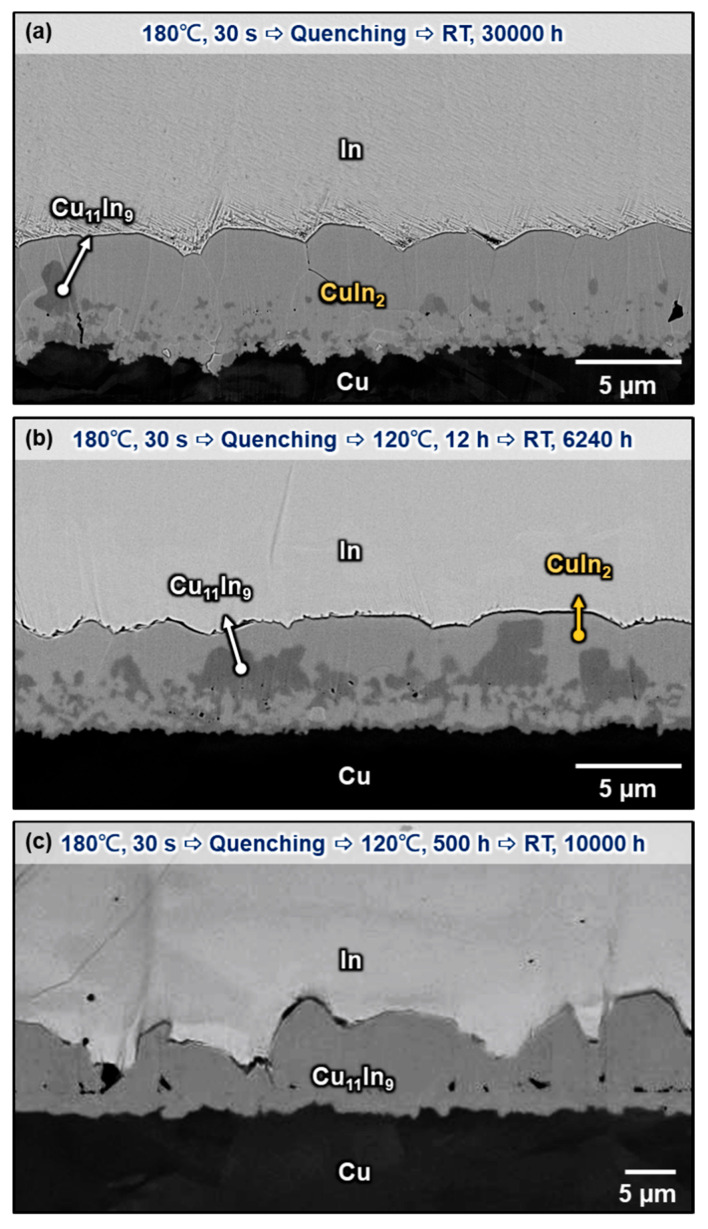
Micrograph showing the Cu/In interfaces after (**a**) storing at room temperature for 30,000 h, (**b**) aging at 120 °C for 12 h and storing at room temperature for 6240 h, and (**c**) aging at 120 °C for 500 h and storing at room temperature for 10,000 h.

In addition, to clearly understand the intermetallic compounds formed between Cu and In at room temperature, three samples with the same soldering conditions (180 °C for 30 s followed by water quenching) and different 120 °C aging periods were introduced. Different aging periods were used to create a Cu/In interface composed of different compositions of compounds. Figure 11a presents the interfacial microstructure of the Cu/In interface after soldering at 180 °C for 30 s and storing at room temperature for 30,000 h. Without 120 °C aging between soldering and room temperature storage, the interfacial structure almost became a continuous CuIn_2_ layer with only some small embedded Cu_11_In_9_ particles after long-term room-temperature storage. The large curvature of the Cu_11_In_9_/In interface in the as-quenched two-phase layer and the relatively low temperature storage at room temperature provide enough driving force for the peritectoid reaction Cu_11_In_9_ + In → CuIn_2_. These results prove that CuIn_2_ is a stable and dominant phase at room temperature. Figure 11b shows the other Cu/In interfacial microstructure, which was aged at 120 °C for 12 h before storage at room temperature for 6240 h. From the previous Section 3.1, we can deduce that CuIn_2_ is not a stable phase at 120 °C, but it needs more than 100 h to decompose at 120 °C. Thus, without a sufficient thermal aging period at 120 °C to consume all CuIn_2_ formed after water quenching, the interfacial structure still became a continuous CuIn_2_ layer with some embedded Cu_11_In_9_ particles after long-term room-temperature storage. Figure 11c presents the interfacial microstructure of the Cu/In interface after 500 h aging at 120 °C plus 10,000 h at room temperature. Compared with Figure 11a,b, it can be seen that the interfacial structure became a continuous Cu_11_In_9_ layer without any CuIn_2_ even after 10,000 h of storage at room temperature. To investigate the difference and realize the intrinsic growth mechanism behind it, the initial condition before room-temperature storage must be considered. As shown in Figure 5f, the 500 h aging period at 120 °C provides a flatter Cu_11_In_9_/In interfacial curvature. Without a sufficiently small curvature to create enough chemical driving force at the Cu_11_In_9_/In interface, there was no formation of CuIn_2_ even after 10,000 h of room temperature storage. In addition, it can be seen that the CuIn_2_ formed in the quenching step has already disappeared completely after aging for 500 h. The lack of CuIn_2_ nucleation restricts the subsequent CuIn_2_ growth. In sum, even though CuIn_2_ is a stable phase at room temperature, without enough interphase curvature between Cu_11_In_9_ and In or any CuIn_2_ residues in Cu_11_In_9_, room temperature storage provides insufficient driving force for the formation of CuIn_2_.

## 4. Conclusions

The microstructure evolution, growth mechanism, and intermetallic identification between solid In and solid Cu at different temperatures were investigated, and the peritectoid temperature range of the Cu_11_In_9_ + In → CuIn_2_ reaction was also acquired in this study. Based on the results obtained, the following conclusions can be drawn:

1. Soldering at 180 ± 1 °C for 30 s was set as the prebonding condition in this study. A two-phase layer consisting of Cu_11_In_9_ and In and faceted Cu_11_In_9_ particles over the two-phase layer formed at the Cu/In interface after the prebonding process.
2. During 120 °C and 140 °C aging, the remaining In in the two-phase layer formed during the prebonding process reacted with the Cu substrate and formed Cu_11_In_9_. Finally, only a continuous Cu_11_In_9_ layer can be observed at the interface.
3. During 100 °C aging, another kind of intermetallic CuIn_2_ formed at the interface. As the aging time increased, Cu_11_In_9_ and In particles in the two-phase layer transformed into CuIn_2_ and finally formed a continuous CuIn_2_ layer. The peritectoid temperature of the Cu_11_In_9_ + In → CuIn_2_ reaction was suggested to be between 100 °C and 120 °C.
4. The place where CuIn_2_ was first observed after quenching is at the Cu_11_In_9_/In two-phase layer, which indicates that a smaller curvature of the Cu_11_In_9_/In interface induces faster CuIn_2_ formation. CuIn_2_ formation at room-temperature storage was observed to be faster than aging at 100 °C, which indicates that a larger temperature difference with the peritectoid temperature induces faster CuIn_2_ formation. The curvature of the Cu_11_In_9_/In interface and the temperature difference with the peritectoid temperature of CuIn_2_ are two factors that influence the growth of CuIn_2_. These two factors influence the driving force of CuIn_2_ formation spontaneously.

## Data Availability

Not applicable.
